# Venous reconstruction in living donor liver transplantation: lessons learned from a new national program in a resource-limited setting

**DOI:** 10.3389/ti.2026.15985

**Published:** 2026-05-04

**Authors:** K. Semash, T. Dzhanbekov, M. Nasirov, A. Subanov, B. Umarov

**Affiliations:** Department of Minimally Invasive Surgery and Transplantation, National Children’s Medical Center, Tashkent, Uzbekistan

**Keywords:** hepatic veins, liver transplantation, living donors, resource-limited settings, venous reconstruction

## Abstract

Complex venous outflow reconstruction in living donor liver transplantation (LDLT) is technically demanding, particularly in resource-limited settings lacking consistent access to synthetic or cryopreserved grafts. We retrospectively analyzed 45 consecutive LDLTs performed during the initiation of a national program. Venous anatomy was evaluated using preoperative CT volumetry and intraoperative findings. Reconstruction strategies included direct anastomosis, unification venoplasty, PTFE grafts, and autologous conduits (falciform ligament, umbilical vein). Outcomes were compared between patients with (n = 17) and without (n = 28) venoplasty. Additional venous reconstruction was required in 37.8% of cases. In 6.7%, anatomically indicated veins could not be reconstructed due to lack of suitable conduits. No early venous thrombosis occurred, and all autologous conduits remained patent during follow-up. Small-for-size physiology developed in 11.1% of recipients, resolved conservatively, and was not associated with unreconstructed major veins. Major morbidity (Clavien–Dindo ≥ IIIb) occurred in 42.2%. The 90-day mortality rate was 11.1%, and 3-year survival was 82.2%, without significant differences between groups. In a newly established program within a resource-limited setting, predominantly autologous venoplasty was feasible and provided satisfactory early and mid-term outcomes.

## Graphical Abstract

**Figure:**
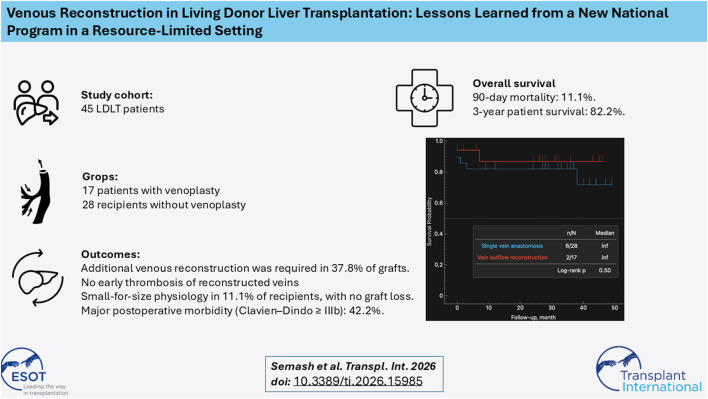

## Introduction

Living donor liver transplantation (LDLT) has become an essential strategy for addressing the persistent shortage of deceased donor organs in many regions of the world. The success of LDLT depends critically on the restoration of adequate venous outflow. Unlike deceased donor liver transplantation that uses a whole organ with relatively uniform venous anatomy, LDLT relies on partial grafts with substantial anatomical variability. This variability creates significant technical challenges during reconstruction of the hepatic veins [1, 2].

Insufficient venous drainage is one of the major determinants of early graft dysfunction. Impaired outflow may result in congestion of the graft, reduction in microvascular perfusion, cholestasis, ischemic injury, and in severe cases complete graft loss [3]. The venous system of the liver demonstrates extensive variation. It includes the right hepatic vein, the middle hepatic vein, the left hepatic vein, and the inferior right hepatic veins. The number, diameter, and drainage patterns of these veins differ markedly among donors. These anatomical factors directly influence the choice of surgical technique required for reconstruction [4–6]. Restoration of tributaries of the middle hepatic vein, accessory veins of segments five and eight, and inferior right hepatic veins becomes especially important when these vessels drain a considerable portion of the graft or when the risk of small for size physiology is anticipated [7–12].

Advances in preoperative imaging, including multidetector computed tomography with three dimensional reconstruction and intraoperative Doppler ultrasonography, provide precise visualization of venous anatomy and allow surgeons to anticipate technical difficulties in advance [13, 14]. These imaging modalities help guide the selection of individualized reconstruction strategies. Depending on the specific configuration of the graft, options may include direct anastomoses, unification venoplasty, extended venoplasty, and the use of autologous, homologous, or synthetic venous conduits [7–11].

Most innovations in venous outflow reconstruction have been developed in the context of right lobe LDLT, where the drainage of segments five and eight presents a particular challenge because the middle hepatic vein is usually retained in the donor to preserve function of the remnant liver. However, left lobe grafts, left lateral sector grafts, and monosegmental grafts also require precise venous reconstruction. In pediatric recipients, and especially in neonates and infants in whom vascular diameters are extremely small, these procedures are frequently performed using microsurgical techniques that reduce the risk of early venous thrombosis [4, 15–18].

Despite considerable progress, there remains no universally accepted standard for venous outflow reconstruction in LDLT. Publications from high volume centers in Japan, the Republic of Korea, China, Taiwan, Singapore, India, and Europe demonstrate substantial variation in surgical approaches [7, 10, 11, 16, 19, 20]. Differences include the criteria for reconstruction of segment five and segment eight tributaries, the preferred type of venous conduit, the method of forming a common venous channel, and the strategies used to maintain patency of reconstructed veins. This variability underlines the need for comprehensive reviews that integrate current evidence with actual clinical experience.

This study incorporates clinical data from our national LDLT program. At present, Uzbekistan has no deceased-donor organ procurement program [21], and all liver transplantations rely exclusively on living related donors, which further restricts the availability of vascular conduits for complex reconstructions. Working within a setting where we had limited availability of prosthetic graft materials and biological conduits, these cases provide important insight into the practical challenges of venous outflow reconstruction. To the best of our knowledge, this study represents the first comprehensive report of venous outflow reconstruction in a newly established national LDLT program operating without a deceased-donor vascular bank and with only intermittent availability of synthetic grafts. This series offers a practical, reproducible framework for other resource-constrained programs worldwide.

## Materials and methods

This study was reviewed and approved by the Institutional Review Board (protocols No 11-17-98/2025 and No 77-77/2025). All procedures were conducted in accordance with the ethical standards of the institutional and national research committees and with the 1964 Helsinki Declaration and its later amendments.

This study was designed as a single-center retrospective observational cohort study and included 45 consecutive living-related donor liver transplantations performed between October 2021 and December 2024. The institutional cases were conducted by the same surgical team and represent the initial phase of program development in a resource limited setting. Overall, 42 cases involved related donorship. The relationships of donors to recipients were as follows: 11 sons, 10 brothers, nine sisters, five cousins, two fathers, two mothers, two aunts, and one nephew. Additionally, under the laws of the Republic of Uzbekistan, spouses are eligible to be organ donors if they have been married for over 3 years [21]. In this study, three wives were approved as donors.

All donors underwent high resolution multiphase computed tomography using a GE Revolution HD 256 slice scanner. A uniform protocol was employed for arterial, portal venous, and hepatic venous phases [21]. The hepatic venous phase served as the primary dataset for evaluation of venous anatomy. Three dimensional reconstruction and volumetric assessment were performed on a GE Advantage Workstation equipped with the Hepatic VCAR application and OsiriX software. In two cases, 3D virtual modeling was used to access vascular anatomy of the donor’s liver (Supplementary video S1) [22].

The venous anatomy was assessed in detail. The right hepatic vein, middle hepatic vein, left hepatic vein, and inferior right hepatic veins were evaluated for number, diameter, distance between their orifices, and drainage territories. Tributaries of segments five and eight were specifically analyzed to determine diameter, length of extrahepatic course, and the angle of entry into the middle hepatic vein. The expected drainage volume of each tributary was estimated using three dimensional volumetry. Reconstruction of S5 and S8 tributaries was planned when at least one of the following objective criteria was met: (1) venous diameter ≥5 mm on preoperative CT or intraoperative measurement; (2) estimated drainage territory ≥20–30% of the anterior sector volume based on three-dimensional volumetry; or (3) graft-to-recipient weight ratio (GRWR) ≤1.0%, indicating increased risk of small-for-size physiology.

Left sided grafts were evaluated as a separate anatomical category. Particular attention was given to the configuration of the left hepatic vein trunk, the drainage patterns of segments two and three, and the relationship between the left hepatic vein and the middle hepatic vein. Left lobe grafts commonly demonstrated separate entry of the middle and left hepatic veins into the inferior vena cava, which necessitated systematic preoperative planning of venous reconstruction. For left lateral segment grafts, venous drainage was reassessed when the segment two and segment three veins entered the inferior vena cava as independent openings. This classification of right sided and left sided drainage patterns guided the selection of unification venoplasty, patch augmentation, or other reconstructive techniques to ensure adequate venous outflow for each graft (Table 1).

**TABLE 1:** Classification of hepatic venous drainage patterns and their implications for reconstruction.

Anatomical group	Key venous features	Diagnostic criteria	Reconstructive implications
Right hepatic vein dominant drainage	Single large RHV draining the majority of the right hemiliver	RHV with sufficient diameter and long extrahepatic cuff	Usually suitable for direct anastomosis. Patch enlargement used when cuff is short
Inferior right hepatic vein dependent drainage	Presence of one or more IRHVs with significant drainage territory	IRHV diameter large enough to require preservation. Visible on CT and confirmed intraoperatively	Often requires separate anastomosis or interposition graft. Failure to reconstruct may lead to segmental congestion
Segment five tributaries (S5)	One or more veins draining segment five toward the MHV	Diameter and drainage area significant on CT	Options include direct anastomosis, unification venoplasty, or patch augmentation depending on number and size
Segment eight tributaries (S8)	Major or minor veins draining segment eight into the MHV	Preoperative CT shows significant S8 venous branch	Frequently requires venoplasty or use of conduits. Combined reconstruction with S5 when orifices are adjacent
Left lobe venous drainage	Separate drainage of MHV and LHV into the inferior vena cava	Consistent finding in majority of left lobe donors. Assessed with three dimensional reconstruction	Venous reconstruction always planned preoperatively due to mandatory restoration of outflow for segments two, three, and four
Left lateral segment drainage (segments two and three)	Segment two and segment three veins draining into IVC through separate openings	Identified on CT and confirmed with 3D reconstruction	Requires unification venoplasty or patch reconstruction to create a common outflow channel and prevent stenosis
Combined right sided and left sided anatomical variants	Complex or mixed drainage patterns involving multiple venous territories	Variability confirmed by preoperative CT + 3D reconstruction	Reconstruction individualized using a combination of direct anastomosis, unification venoplasty, patch augmentation, or conduits

Abbreviations: RHV, right hepatic vein; MHV, middle hepatic vein; LHV, left hepatic vein; IRHV, inferior right hepatic vein; S5, segment five; S8, segment eight; S2, segment two; S3, segment three; IVC, inferior vena cava.

The surgical technique for donors and recipients has been described previously in our previous publications [23]. Intraoperatively, the diameters of the S5, S8, and inferior right hepatic veins were assessed, and veins measuring greater than 5 mm were classified as significant; in addition, back-table evaluation included assessment of preservative solution outflow through individual venous branches, and veins measuring 5 mm or less were also considered for reconstruction when they demonstrated substantial efflux of preservative solution. Reconstruction of inferior right hepatic veins was performed by direct end-to-side anastomosis to the recipient inferior vena cava. Each significant iRHV was implanted separately when anatomically feasible to ensure adequate venous drainage and prevent segmental congestion. In cases where an isolated significant segment V (S5) or segment VIII (S8) tributary was identified, the respective vein was reconstructed using an interposition conduit and anastomosed to the recipient middle hepatic vein to restore anterior sector outflow. When both S5 and S8 tributaries were deemed significant, reconstruction of middle hepatic vein performed on the graft, which was then reconstructed toward the recipient IVC. If necessary, Unification plasty was performed using a continuous 6/0 polydioxanone suture. When autologous conduits (falciform ligament or recipient umbilical vein) were utilized, reconstruction of the corresponding venous branches was carried out on the back table. The conduit was tailored to the required diameter, anastomosed to the segmental veins under magnification, and tested for watertight integrity using preservation solution before implantation. After graft implantation, venous outflow patency was monitored with Doppler ultrasonography immediately after reperfusion, at twenty-four hours, and at regular intervals during the postoperative period. Computed tomography was obtained in cases where ultrasonographic evaluation suggested impaired venous flow. Laboratory markers and clinical parameters of graft congestion were correlated with imaging findings. We also evaluated postoperative criteria for small for size syndrome. Clinical and laboratory indicators were monitored in accordance with established definitions of small for size physiology, including early hyperbilirubinemia, coagulopathy that persisted despite correction, excessive ascites production, and characteristic trajectories of aminotransferase levels [24]. Hemodynamic parameters such as portal venous flow and pressure were assessed when clinically indicated. These variables were analyzed to identify early signs of graft dysfunction related to insufficient graft size or compromised venous outflow.

Follow-up was conducted from the date of transplantation until the last available clinical assessment. The duration of follow-up varied among patients due to the ongoing nature of the program.

Continuous variables were reported as mean ± standard deviation or median [interquartile range], according to their distribution, which was assessed using the Shapiro–Wilk test. Categorical variables were summarized as absolute and relative frequencies. Group comparisons (venoplasty vs. no venoplasty) were performed using Fisher’s exact test or the chi-square test for categorical variables and Student’s t-test or the Mann–Whitney U test for continuous variables, selected based on data distribution and variance homogeneity. Potential confounders were evaluated through univariable analyses, and variables with clinical relevance or p < 0.10 were entered into a multivariable logistic regression model to identify independent predictors of early biliary leakage. Adjusted odds ratios with 95% confidence intervals were calculated, and model fit was assessed using standard goodness-of-fit diagnostics. Survival outcomes were estimated using the Kaplan–Meier method with right-censoring at the date of last follow-up. A *post hoc* exploratory subgroup analysis was performed comparing the first 30 cases with the last 15 cases to assess the potential influence of program maturation and learning curve on outcomes. A two-sided p-value <0.05 was considered statistically significant. All analyses were conducted using IBM SPSS Statistics, version 26.0 (IBM Corp., USA).

## Results

A total of forty-five grafts were analyzed, including forty-two right lobe grafts, one left lobe grafts, and two left lateral segment grafts. Variants of hepatic venous anatomy and the corresponding reconstructive strategies are summarized in Table 2. Median GRWR was 1.1% (0.7-2.7). The median GRWR was 1.05% (range 0.7%–2.0%) in the venoplasty group and 1.1% (range 0.85%–2.7%) in the no-venoplasty group.

**TABLE 2:** Venous Outflow Anatomy and Reconstruction Techniques (n = 45 grafts).

Graft type	Venous structure/configuration	Number of grafts	Reconstruction/anastomosis technique
RL	Single RHV, no significant accessory veins or there was no possibility of reconstruction	28	Single caval anastomosis; no venoplasty
RL	One additional iRHV	3	Two caval anastomoses; no venoplasty
RL	Three RHVs (two iRHV accessory veins)	3	Unification plasty of iRHVs + two caval anastomoses
RL	PTFE graft to S5 vein	2	PTFE conduit anastomosed to IVC
RL	PTFE graft to S8 vein	1	PTFE conduit anastomosed to IVC
RL	PTFE graft connecting S5 + S8 and separate iRHV	1	PTFE conduit of separate S5 and S8 → three anastomoses with IVC
RL	S5+S8 reconstruction using falciform ligament graft	2	Autologous FL conduit for unification, anastomosed to IVC
RL	S5+S8 reconstruction using umbilical vein of recipient	1	Umbilical vein conduit obtained from recipient for unification, anastomosed to IVC
RL	S8 was close to the RHV	1	Unification plasty of RHV and S8
LL	Three separate veins (MHV, S2, S3)	1	Unification plasty
LLS	Single LHV	1	Single caval anastomosis; no venoplasty
LLS	Separate S2 and S3 veins	1	Unification plasty

Abbreviations: RL, right lobe; LL, left lobe; LLS, left lateral section; RHV, right hepatic vein; MHV, middle hepatic vein; LHV, left hepatic vein; iRHV, inferior right hepatic vein.

S2, S3, S5, S8, hepatic segmental veins corresponding to Couinaud segments two, three, five, and eight; IVC, inferior vena cava; PTFE, polytetrafluoroethylene; FL, falciform ligament.

### Right liver grafts

**FIGURE 1:**
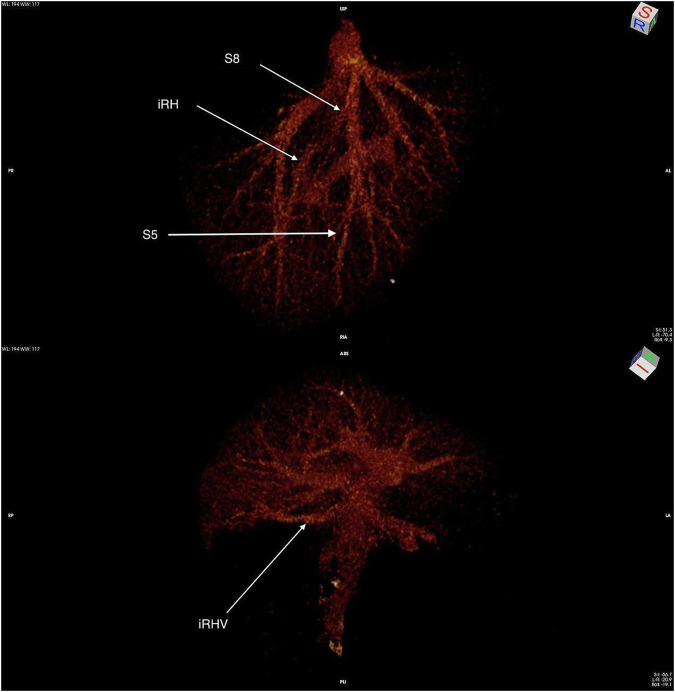
Three-dimensional CT reconstruction of the donor’s venous outflow. Arrows indicate the significant venous branches of segments 5 and 8, as well as the inferior right hepatic vein (IRHV).

The majority of right liver grafts demonstrated a single dominant right hepatic vein. In twenty eight cases, a single right hepatic vein with no significant accessory branches was present, which allowed for a single caval anastomosis without the need for venoplasty. When a hepatic vein that required reconstruction was left unreconstructed, post-reperfusion inspection consistently revealed an area of congested graft parenchyma with a darker, bluish discoloration (Figure 1).

An additional inferior right hepatic vein was identified in three grafts. In these cases, two separate caval anastomoses were performed, and no venoplasty was required because the diameter and venous territory of the inferior right hepatic vein permitted direct implantation.

Two grafts demonstrated three independent right hepatic venous trunks, two of which corresponded to inferior right hepatic veins with significant drainage territories. These cases required unification venoplasty of the inferior right hepatic veins followed by two separate caval anastomoses.

**FIGURE 2:**
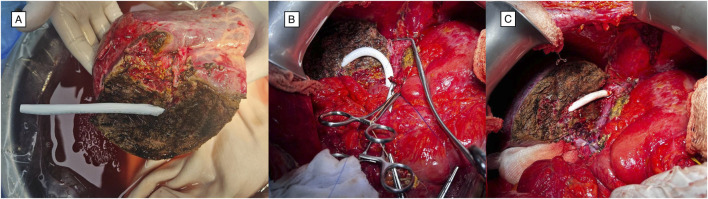
Reconstruction of segment five vein in right lobe liver graft using PTFE graft. **(A)** Back-table stage. A PTFE conduit was anastomosed with significant S5 vein on the liver graft. **(B)** A veno-caval anastomosis was performed. Preparation for PTFE graft anastomosis. **(C)** The PTFE conduit was anastomosed to the recipient’s middle hepatic vein. Appearance after venous reperfusion. Abbreviations: PTFE, polytetrafluoroethylene; S5, segment V of the Couinaud liver segmentation system.

**FIGURE 3:**
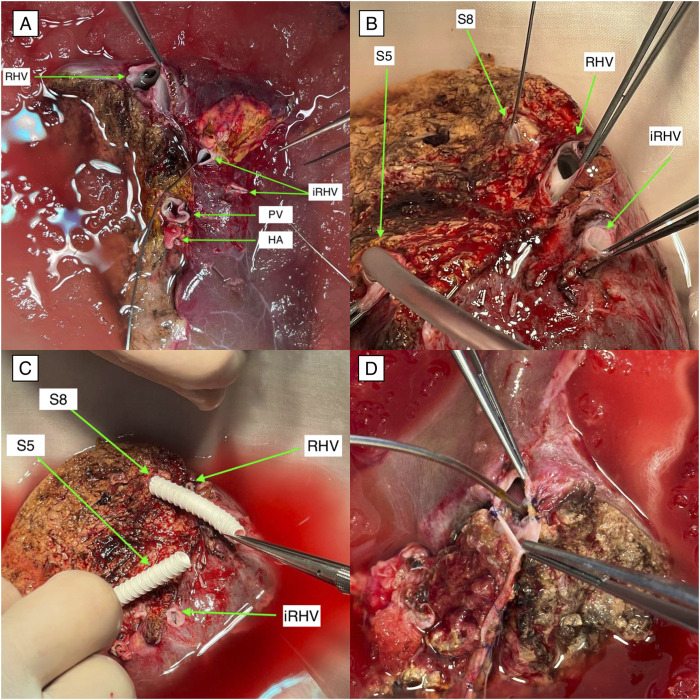
Variations of venous anatomy in liver grafts **(A)** A right-lobe liver graft with two separate inferior right hepatic veins required unification venoplasty of the inferior veins and construction of two distinct veno-caval anastomoses. **(B)** A right-lobe liver graft with significant venous branches from segments 5 and 8, as well as an additional inferior right hepatic vein. S5 vein canulated for conservant solution outflow assession. **(C)** A venous reconstruction approach for segments 5 and 8 using separate PTFE conduits; additionally, this graft contained an accessory inferior right hepatic vein that required an independent anastomosis to the inferior vena cava. **(D)** A left-lobe liver graft in which unification venoplasty was performed to combine the S2 and S3 venous branches with the middle hepatic vein; the probe is positioned within the lumen of the S3 vein. Abbreviations: PTFE, polytetrafluoroethylene; S2, S3, S5, S8, segments II, III, V, and VIII of the Couinaud liver segmentation system.

Reconstruction of segmental veins from segments five and eight was required in several grafts. PTFE interposition grafts were used in five grafts. In two cases a PTFE conduit was used for outflow reconstruction of a segment five vein, and in one of these cases conduit was anastomosed to the recipient’s middle hepatic vein draining into the inferior vena cava (Figure 2). One graft required PTFE reconstruction for a segment eight vein. In one graft a single PTFE conduit was used to unify and drain both segment five and segment eight veins into the inferior vena cava. Additionally, in one case PTFE conduits were used to reconstruct the separate S5 and S8 hepatic veins, resulting in three distinct outflow channels that were anastomosed individually to the IVC (Figures 3A–C).

**FIGURE 4:**
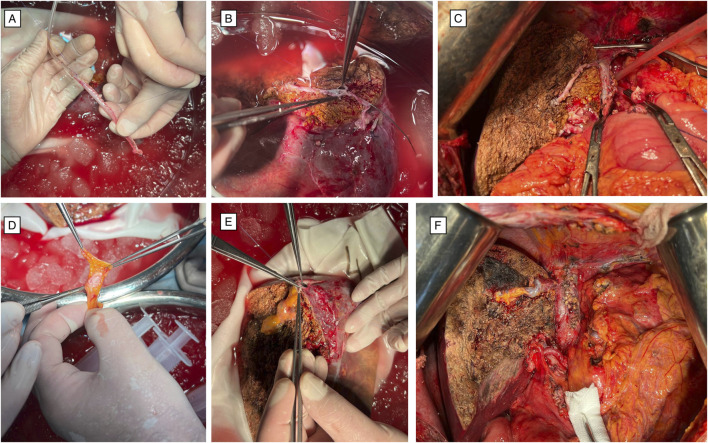
**(A)** A conduit was fashioned on the back table from the donor’s falciform ligament, which was wrapped around a sterile 14-F tube to create a tubular graft. **(B)** Anastomosis of the falciform-ligament conduit to the venous branches of segments 5 and 8 was performed and unified with the right hepatic vein on the graft. **(C)** Appearance of the graft prior to venous reperfusion. **(D)** The recipient’s umbilical vein shunt was harvested, and openings were created to enable anastomosis with the significant venous branches of segments 5 and 8. **(E)** The umbilical vein conduit was anastomosed to the S5 and S8 branches and unified with the right hepatic vein on the graft. **(F)** Appearance of the graft following venous reperfusion. Abbreviations: PTFE, polytetrafluoroethylene; S5, S8, segments V, and VIII of the Couinaud liver segmentation system.

Autologous tissue was used for venous reconstruction in three cases. Two grafts required unification of the segment five and segment eight veins using a conduit fashioned from the falciform ligament (Figures 4A–C). In one case the umbilical vein of the recipient was harvested and used as an interposition conduit to reconstruct the outflow of both segment five and segment eight veins (Figures 4D–F).

One graft demonstrated a segment eight tributary running very close to the right hepatic vein. In this case, unification venoplasty was performed without the use of interpositional material.

### Left liver grafts

During harvesting LL graft, we faced an unusual and technically challenging venous configuration that resulted directly from an intraoperative mechanical complication. During donor left hepatectomy, the stapling device malfunctioned, causing the venous structures to be transected lower than the planned level. As a result, the middle hepatic vein, the segment two vein, and the segment three vein were obtained as three separate orifices located at a considerable distance from one another. The distance-spaced venous openings required an extensive and complex unification venoplasty to create a single, adequate outflow channel suitable for implantation into the inferior vena cava (Figure 3D).

Also, two left lateral section grafts were included. One graft contained a single left hepatic vein and required only one caval anastomosis without the need for additional venoplasty. The second graft demonstrated separate venous orifices from segment two and segment three. These veins were reconstructed using unification venoplasty.

### Postoperative outcomes

Early and late postoperative complications were compared between recipients who underwent a single hepatic vein anastomosis (no venoplasty, n = 28) and those who received venous outflow reconstruction (venoplasty, n = 17) (Table 3). Overall, most vascular complication rates were low and did not differ significantly between the groups.

**TABLE 3:** Postoperative outcomes.

Parameter	No venoplasty (n = 28)	Venoplasty (n = 17)	p-value
Hepatic artery thrombosis	1 (3.6%)	0	1.000
Hepatic artery stenosis	3 (10.7%)	1 (5.9%)	1.000
Splenic artery steal syndrome	3 (10.7%)	0	0.290
Portal vein thrombosis	1 (3.6%)	2 (11.8%)	0.546
Postoperative bleeding requiring relaparotomy	2 (7.1%)	0	0.525
Clinical features for small-for-size syndrome	3 (10.7%)	2 (11.8%)	1.000
Early biliary leak — overall	3 (10.7%)	10 (58.8%)	0.001
Early biliary leak — first 30 cases (1–30)	3/16 (18.8%)	9/14 (64.3%)	0.023
Early biliary leak — last 15 cases (31–45)	1/12 (8.3%)	0/3	1.000
Late biliary stricture	1 (3.6%)	1 (5.9%)	1.000
Number of bile duct orifices — mean ± SD	1.8 ± 0.8	2.4 ± 0.7	0.018
Number of biliary anastomoses — mean ± SD	1.7 ± 0.7	2.4 ± 0.6	0.006
Clavien–Dindo ≥ IIIb	10 (35.7%)	9 (52.9%)	0.360
90-day mortality	3 (10.7%)	2 (11.8%)	1.000
Comprehensive complication index, median [IQR]	20.9 [0–39.8]	42.6 [26.2–66.3]	0.032
Patient actuarial survival 1/3 years	86.8%/82.8%	87.5%/81.2%	0.78

Hepatic artery thrombosis occurred only in the single-vein group (3.6%) and was not observed in venous reconstruction group (p = 1.000). Hepatic artery stenosis was comparable between cohorts (10.7% vs. 5.9%, p = 1.000), with a non-significant trend toward a lower risk in the venoplasty group (OR 0.58, 95% CI 0.06–6.06). Clinical features consistent with splenic artery steal syndrome (SASS) were observed only in patients without venoplasty (10.3% vs. 0%, p = 0.542). SASS was defined clinically as a combination of persistently reduced hepatic arterial flow on Doppler ultrasound in the absence of hepatic artery thrombosis or stenosis, associated with a disproportionately increased splenic artery flow, and improvement after splenic artery–directed intervention or conservative management. Angiography was performed selectively when clinically indicated.

Portal vein thrombosis developed more frequently among patients who underwent venous reconstruction (11.8% vs. 3.6%), although the difference did not reach statistical significance (p = 0.287; OR 4.00, 95% CI 0.34–47.4).

Early biliary leakage occurred in 13 of 45 patients (28.9%). The incidence was significantly higher in the venoplasty group (58.8% vs. 10.7%, p = 0.001). This difference was most pronounced during the first 30 cases (64.3% vs. 18.8%, p = 0.023) and decreased in the last 15 consecutive cases after introduction of an ultrasonic cavitation aspirator (CUSA), improved liver retraction systems, and stricter donor selection (8.3% vs. 0%, p = 1.000). Multivariable logistic regression showed that venoplasty was not an independent predictor of early biliary leak (adjusted OR 2.8, 95% CI 0.6–13.2, p = 0.19). The only independent risk factors were the number of bile duct orifices (OR 4.7 per additional orifice, 95% CI 1.6–13.8, p = 0.005) and the early phase of the program (first 30 vs. last 15 cases: OR 22.4, 95% CI 2.5–201, p = 0.005). Late biliary strictures occurred in two patients (4.4%), one in each group, and were successfully managed surgically.

Postoperative bleeding requiring relaparotomy was noted only in the no-venoplasty cohort (6.9% vs. 0%, p = 0.531). The overall burden of severe complications (Clavien–Dindo ≥ IIIb) was higher among patients who underwent venous reconstruction (50.0% vs. 34.5%), although this difference was not statistically significant (p = 0.360; OR 1.90, 95% CI 0.56–6.45). 90-day mortality (Clavien–Dindo grade V) was similar between groups (12.5% vs. 10.3%, p = 1.000). The Comprehensive Complication Index (CCI) demonstrated a significantly higher cumulative morbidity in the venoplasty group, with a median of 39.8 (IQR 26.2–66.3) compared with 20.9 (IQR 0–39.8) in the single-vein cohort (p = 0.047), indicating a greater severity and overall burden of complications in patients requiring venous reconstruction.

Clinical features consistent with small-for-size syndrome were observed in five recipients (11.1%). Three cases occurred in the no-venoplasty group (3/28, 10.7%) and two in the venoplasty group (2/17, 11.8%), with no statistically significant difference between groups (p = 1.000). SFSS was defined clinically based on persistent hyperbilirubinemia, refractory ascites, and/or prolonged coagulopathy in the absence of mechanical outflow obstruction or other identifiable causes. Management was performed according to standard recommendations and included optimization of hemodynamics, albumin supplementation, diuretic therapy, careful fluid balance control, and close Doppler surveillance. All cases were managed conservatively without graft loss attributable to SFSS.

There was no venous outflow–related complications in the study cohort. No cases of early thrombosis of reconstructed venous outflow were observed. Refractory ascites was observed in 3 patients (6.7%) and was attributed to small-for-size syndrome, as there was no objective evidence of impaired venous outflow in these patients, in conjunction with a low graft-to-recipient weight ratio.

**FIGURE 5:**
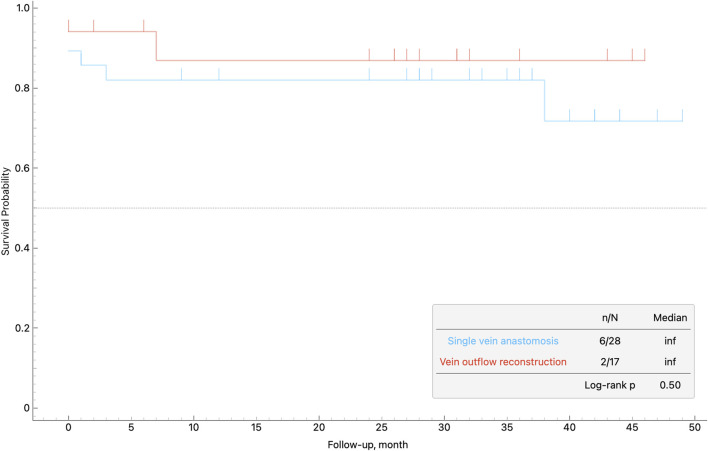
The actuarial Kaplan–Meier overall patient survival curves stratified by performance of venoplasty.

The median follow-up duration was 32.5 months for patients without venoplasty and 27 months for those who underwent venoplasty. Across the entire cohort, the median follow-up time was 29 months. Overall patient survival did not differ significantly between recipients who underwent venoplasty and those with a single vein anastomosis (log-rank p = 0.62; Figure 5). The actuarial 1-year and 3-year patient survival rates estimated by Kaplan–Meier analysis were were 86.8% and 82.8%, in the single-anastomosis group and 87.5% and 81.2% in the reconstruction group, respectively. Median survival was not reached in either group during the maximum follow-up of 50 months. Hospital mortality in the cohort was 11.1% (5 of 45 patients). Two patients died from severe acute rejection, two from sepsis, and one from complications related to portal vein thrombosis. During long-term follow-up, three additional deaths occurred: one due to COVID-19 associated pneumonia, one due to nonadherence to immunosuppressive therapy, and one due to aspiration. No death in the entire series was attributable to venous outflow compromise.

## Discussion

Venous outflow reconstruction remains one of the principal determinants of graft function in living donor liver transplantation [11]. The present series illustrates not only the anatomical complexity of partial liver grafts but also the considerable constraints imposed by a resource-limited environment. The absence of a deceased donor program, the restricted availability of vascular prostheses, and the inability to perform graft biopsy for differential diagnosis significantly influenced surgical decision-making and postoperative management. These conditions differentiate our experience from that of high-volume centers in East Asia and Europe, where venous reconstruction techniques have evolved in settings with broad access to vascular materials and advanced diagnostic infrastructure.

At the initial stage of the program, donor selection was intentionally conservative: we prioritized donors with straightforward hepatic anatomy and grafts with a favorable graft-to-recipient weight ratio. As surgical experience accumulated, we gradually expanded the range of acceptable anatomical variations and began to undertake more complex venous outflow reconstructions. International experience consistently supports an assertive strategy for reconstruction of segment V and segment VIII tributaries when these veins exceed four or 5 mm in diameter or drain a substantial portion of the anterior sector [11, 25]. This approach is grounded in extensive evidence from different transplant centers, where centers routinely employ autologous, homologous, and synthetic conduits and where vascular banking is standard practice. Studies by Sakamoto, Jeng, Thorat, Taha, Pamecha, Park, and others demonstrate that restoration of middle hepatic vein tributaries improves parenchymal perfusion, reduces venous congestion, enhances early graft regeneration, and mitigates the risk of small-for-size physiology [7, 8, 12, 20, 25–27]. Work by Mizuno et al has shown that regeneration of the anterior sector is significantly greater in grafts with reconstructed venous outflow [28].

The application of these principles in our program was necessarily selective. Although preoperative imaging and intraoperative assessment in several cases demonstrated clear indications for venous reconstruction, the unavailability of PTFE grafts in these cases made standard reconstruction technically impossible. Decisions were therefore individualized. Reconstruction was prioritized when volumetric analysis indicated a substantial drainage territory, when diameters exceeded 5 mm, and when the graft-to-recipient weight ratio approached the lower acceptable threshold. When prosthetic material was unavailable, alternative methods were employed. These included reconstruction with the falciform ligament, the recipient’s umbilical vein, or spontaneously developed portosystemic shunts. Such solutions reflect the adaptive strategies described in early LDLT program development and demonstrate that acceptable venous outflow can be achieved even in the absence of dedicated vascular grafts.

An additional aspect that deserves consideration is the use of venous patch augmentation, which is frequently employed in high-volume transplant centers to facilitate creation of a wide and congruent anastomotic surface. Patch reconstruction enlarges the venous orifice, reduces the risk of anastomotic stenosis, and improves the geometric alignment between the graft vein and the recipient inferior vena cava. In many programs, homologous venous patches obtained from deceased donors or synthetic prosthetic patches are routinely applied [29]. In our series, patch venoplasty was not performed because no deceased-donor venous patches were available in our setting.

The most notable difference between the groups was the higher crude incidence of early biliary leaks in patients who underwent venoplasty (58.8% vs. 10.7%, p = 0.001). However, this association was almost entirely confined to the initial phase of the program and disappeared completely after technical maturation. Of the 13 early biliary leaks, 12 (92.3%) occurred within the first 30 cases (64.3% vs. 18.8% in the venoplasty and no-venoplasty groups, respectively). In the most recent 15 consecutive LDLTs (cases 31–45), performed after introduction of an ultrasonic cavitation aspirator (CUSA), advanced liver retraction systems, and stricter donor selection, only one early biliary leak was recorded in the entire cohort (6.7%), and it occurred in the no-venoplasty group (0% vs. 8.3%, p = 1.000). Multivariable logistic regression confirmed that venoplasty was not an independent risk factor for biliary leakage (adjusted OR 3.1, 95% CI 0.6–15.4, p = 0.17). The only independent predictors were multiple bile duct orifices (OR 4.9 per additional orifice, 95% CI 1.6–15.0, p = 0.005) which were significantly more common in grafts requiring venous reconstruction and the early program period (first 30 vs. last 15 cases: OR 24.8, 95% CI 2.7–227, p = 0.004). These findings strongly indicate that the observed association was driven by selection of anatomically complex donors and the institutional learning curve rather than by the venous reconstruction itself. In the mature phase of the program, venoplasty was not associated with increased biliary morbidity. Collectively, these factors, together with the influence of the institutional learning curve [30], likely affected the outcomes observed during the early development of our program. Our biliary leak rates align with reported ranges in emerging LDLT programs [31], and the rapid decline demonstrates the impact of technical evolution.

A further challenge of the resource-limited setting was the absence of liver biopsy, which prevented histological differentiation of early postoperative dysfunction. Biopsy remains the most reliable method to distinguish small-for-size syndrome from acute rejection and sepsis-associated cholestasis [32, 33]. In our series, clinical assessment depended on biochemical trends, Doppler ultrasound, and advanced imaging. Clinical suspicion of small for size syndrome was documented in five recipients. Three of these patients presented exclusively with high volume ascitic output during the first ten postoperative days. Daily drainage volumes reached up to four thousand milliliters and required replacement with crystalloid solutions, albumin infusion, and prolonged maintenance of abdominal drains. Two of these three patients had a single venous anastomosis and relatively small grafts with GRWRs of 0.7 and 1.1. The third patient with the same clinical pattern had two venous anastomoses, specifically the right hepatic vein and an inferior right hepatic vein, and a GRWR 1.1. The clinical pattern was consistent with the diagnostic framework proposed by the ILTS consensus group [34, 35]. In another patient, uncontrolled hyperbilirubinemia initially raised concern for outflow impairment, but the clinical course ultimately revealed reactivation of autoimmune hepatitis, which responded favorably to bortezomib [36]. This case underscores the diagnostic uncertainty inherent in the absence of histological confirmation. One female recipient with a GRWR of 0.9 and a single venous outflow presented with uncontrolled postoperative hyperbilirubinemia. Mechanical obstruction was excluded by magnetic resonance cholangiopancreatography and endoscopic retrograde cholangiopancreatography. The clinical picture was interpreted as a combination of sepsis and acute rejection. Due to limited resources, graft biopsy could not be performed. On postoperative day five, the patient developed ovarian apoplexy which required surgical intervention. Postoperative infectious complications progressed, and the patient ultimately died from sepsis.

The observation that clinical features suggestive of splenic artery steal syndrome occurred only in grafts without venoplasty should be interpreted cautiously. Given the retrospective design and limited number of events, this finding is hypothesis-generating and does not imply a causal protective effect of venous reconstruction.

Donor morbidity was analyzed according to the Clavien–Dindo classification. Overall, complications were predominantly minor and managed conservatively. Wound seroma developed in two donors and was treated with local measures. One donor developed hospital-acquired pneumonia (Clavien–Dindo grade II), which resolved with appropriate medical treatment. One donor developed acute renal dysfunction during antibacterial prophylaxis with sulperazone, presenting with oliguria, proteinuria, hematuria, peripheral edema, and pleural effusion. Sulperazone was discontinued, and diuretic therapy was initiated, resulting in complete recovery of renal function. Pleural effusion occurred in two donors and required therapeutic thoracentesis. Two donors developed postoperative bilomas that were successfully managed with percutaneous drainage. In two additional cases of bile leakage, surgical revision was necessary. One donor experienced postoperative bleeding due to slippage of a clip from the inferior vena cava, necessitating urgent reoperation. No donor mortality occurred in this series.

Comparison of our findings with the international literature reveals considerable alignment. The functional importance of anterior sector drainage, the necessity for detailed preoperative three-dimensional venous mapping, and the contribution of reconstructed middle hepatic vein tributaries to early graft performance are all consistent with established evidence [17, 34]. Our results also emphasize that acceptable outcomes can be achieved in a resource-constrained program when surgical planning is meticulous and techniques are adapted to the available materials. In cases with a high GRWR, the physiological reserve of the graft may compensate for small unreconstructed venous branches, and reconstruction is not always necessary or feasible. The frequent need to substitute autologous or unconventional conduits for synthetic or homologous grafts highlights the divergence between theoretically optimal strategies and the realities of clinical practice in a developing transplantation system.

Several limitations of the present study stem directly from the context in which the program operates. The absence of a deceased donor program precludes procurement and cryopreservation of vascular conduits. The intermittent unavailability and high cost of PTFE grafts restrict their use and limit uniformity of surgical technique. The lack of biopsy capability complicates postoperative diagnostic accuracy. In addition, the proportion of left lobe and left lateral segment grafts was small. Also, donor selection during the initial period was intentionally conservative, favoring anatomically straightforward grafts with favorable GRWR, which may introduce selection bias. In addition, the progressive accumulation of surgical experience and technical refinements over time represent potential confounding factors that may have influenced complication rates independently of venous reconstruction strategy. Therefore, subgroup comparisons should be interpreted cautiously. Despite these limitations, this series represents the early developmental phase of a national LDLT program and illustrates the feasibility of venous outflow reconstruction under resource-constrained conditions, while underscoring the importance of structured program maturation and cautious interpretation of outcomes.

## Conclusion

This study demonstrates that venous outflow reconstruction in living donor liver transplantation can be successfully implemented in a resource-limited setting during the early phase of program development. In this cohort, autologous reconstruction techniques provided satisfactory early and mid-term patency without evidence of venous outflow–related graft loss.

Importantly, our experience indicates that clinical outcomes were influenced not only by the reconstructive strategy itself but also by progressive institutional learning, refinement of surgical technique, and more selective donor assessment. The marked reduction in biliary complications over time highlights the critical role of programmatic maturation in determining results.

These findings suggest that, in emerging transplant programs, structured technical standardization and accumulation of surgical experience may be as important as the choice of venous reconstruction method in achieving safe and reproducible outcomes. Further studies with larger cohorts and longer follow-up are required to confirm long-term durability.

## Data Availability

The raw data supporting the conclusions of this article will be made available by the authors, without undue reservation.

## Supplementary material

SUPPLEMENTARY VIDEO S1 Three-dimensional reconstruction of the hepatic vasculature in a living donor using SurgiPrint software. The use of 3D imaging enables a detailed assessment of vascular anatomy and spatial relationships between vessels, thereby facilitating precise preoperative surgical planning.
